# Lexical pathway from L2 to L1 activation in intermediate proficient bilinguals: behavioral and ERP evidence

**DOI:** 10.3389/fnhum.2024.1270377

**Published:** 2024-06-03

**Authors:** Siqin Yang, Siyi Jiang, Minghu Jiang, Qian Guo

**Affiliations:** ^1^School of Liberal Arts, Renmin University of China, Beijing, China; ^2^Beijing Key Laboratory of Applied Experimental Psychology, Faculty of Psychology, Beijing Normal University, Beijing, China; ^3^Center for Psychology and Cognitive Science, Tsinghua University, Beijing, China; ^4^Department of Foreign Languages and Literatures, Tsinghua University, Beijing, China

**Keywords:** bilingual, cross-language, activation, visual-word recognition, pathway, Mandarin–English

## Abstract

Numerous studies have demonstrated that second language (L2) comprehension is often accompanied by activations in the first language (L1). Using both behavioral measurement and event-related potential (ERP), this study conducted two experiments to investigate whether a direct activation pathway exists from L2 lexical representation to L1 lexical representation (the lexical pathway) in intermediate proficient bilinguals. In Experiment 1, we designed a vowel letter search task on English word pairs, which enables bilinguals to prevent semantic priming in the first 300 ms processing stage after the words’ onset. In Experiment 2, Mandarin–English bilinguals were recruited to complete this task on English word pairs with occasional first character repetition between the Chinese counterparts of a word pair. Results showed a significant main effect within both the P200 and N400 time windows, indicating the activation of bilinguals’ L1 lexical representation during these intervals. However, the main effect of semantic relatedness was only significant in the N400 time window. These results suggest that bilinguals can activate their L1 lexical representation directly before engaging in conceptual representation. This finding supported a lexical pathway of activation from L2 lexical representation to L1 lexical representation during visual-word recognition in intermediate proficient bilinguals.

## 1 Introduction

Cross-language activation in bilinguals has emerged over the past few decades as a prominent topic in psychology, linguistics, and neuroscience (Caramazza and Brones, 1979; Wu and Thierry, 2012; Kapnoula and Samuel, 2019; Ma et al., 2020). Numerous studies have shed light on the phenomenon of first-language (L1) word activation during second-language (L2) word comprehension. The aim of the present study was to examine the lexical pathway of activation from L2 lexical representation to L1 lexical representation during visual-word recognition in intermediate proficient Mandarin–English bilinguals. Combined with bilinguals at different L2 proficiency levels, the result of the current research may provide critical insights into the theoretical underpinnings of the mental lexicons of bilinguals.

Several empirical studies have consistently demonstrated that bilinguals can automatically activate their L1 when engaged in semantically related tasks (e.g., semantic relatedness judgment task and animacy decision task) within their L2 context (Thierry and Wu, 2004; Thierry and Wu, 2007; Wu and Thierry, 2010; Wang et al., 2017). To illustrate, in the Wu and Thierry (2010) study, Mandarin–English bilingual participants performed a semantic relatedness judgment task involving L2 word pairs [e.g., train (火车in Chinese) and ham (火腿 in Chinese)]. They were found to be able to perceive the sound repetition in the Chinese counterparts of the L2 word pairs [e.g., 火 (huo3)]. These findings are commonly interpreted within the conceptual framework of either the Bilingual Model of Lexical Access (BIMOLA) (Spivey and Marian, 1999) or the Bilingual Interactive Activation Model Plus (BIA+) (Dijkstra and van Heuven, 2002). Both models propose a comprehensive, interconnected identification system and posit that lexical representation of the non-target language can be activated non-selectively.

With respect to the intricate network of cross-language activation pathways that may potentially underlie the recognition of words, another bilingual mental lexicon, the Revised Hierarchical Model of Lexical and Conceptual Representation in Bilingual Memory (referred to as the “Revised Hierarchical Model” or RHM), which assumes the conceptual representation is shared while the lexical representation is separate, describes both indirect and direct connections between L1 lexical representation and L2 lexical representation (Kroll and Stewart, 1994; Kroll et al., 2010; Clenton, 2015). The indirect cross-language connection is achieved through conceptual representation. The direct cross-language connection is accomplished directly between lexical representations across languages. In the present study, we define the conceptual pathway as the indirect cross-language connection and the lexical pathway as the direct cross-language connection.

For visual-word recognition, several studies provided compelling evidence regarding the activation of the first language (L1) through the conceptual pathway while bilinguals were engaged in semantically related tasks in their second language (L2) (Thierry and Wu, 2004; Thierry and Wu, 2007; Wu and Thierry, 2010; Wu and Thierry, 2012; Khachatryan et al., 2016). It was found that only if they invoked the conceptual representation could they then make correct judgments about semantic relatedness. Furthermore, studies found that regardless of bilinguals’ L2 level and the language in which they perform semantically related tasks, their conceptual representation is activated (Yang et al., 2021a,b). These research results suggest the existence of a conceptual pathway for cross-language activation from L2 lexical representation to L1 lexical representation during visual-word recognition by bilinguals at different L2 levels.

In contrast, the activation from L2 to L1 lexical representations through the lexical pathway for visual-word recognition in different studies seemed to vary for bilinguals with different L2 levels. Some scholars claimed that bilinguals with low proficiency could activate their conceptual representation of the L2 lexical representation only by activating their corresponding L1 lexical representation. In their study, Guo and Peng (2003) invited bilinguals with low proficiency to perform a cross-language priming task. The results showed that the Chinese target word (e.g., 杯, glass in English) would be primed by the English word whose Chinese translation was similar to the target word (e.g., bad, 坏 in Chinese) as the two Chinese words share a common radical. However, when these two Chinese words were only semantically related, without such orthographic similarity such priming effects disappeared. The researcher interpreted this phenomenon as indicating that when bilinguals read English words, their Chinese counterparts were directly activated. Such direct cross-language activation was also observed in Mandarin–English bilinguals who were non-English majors and did not pass the College English Test Band 4 (CET-4), which is the lowest band of CET (Li et al., 2009). These results were consistent with the RHM conceptual framework, which assumes that bilinguals with low L2 proficiency can directly activate their L1 lexical representation from the L2 lexical representation.

Unlike low-proficiency bilinguals, high-proficiency bilinguals have been found to activate their conceptual representation directly without activating their L1 lexical representation. In Mo et al.’s (2005) experiment, when Mandarin–English bilinguals who majored in English performed a long-term cross-language repetition priming task, the researchers failed to find any cross-language activation between L1 lexical representation and L2 lexical representation in a lexical decision task. A similar result was also obtained in a study with Dutch–English bilinguals proficient in English (Zeelenberg and Pecher, 2003). These results were also in accordance with predictions from the RHM regarding higher L2 proficiency. Specifically, the RHM proposes that as L2 use and proficiency increase, the connection between L2 lexical representation and conceptual representation and conceptual representations are strengthened. Meanwhile, the direct connection between L1 lexical representation and L2 lexical representation may progressively weaken or disappear entirely. Bilinguals may directly activate conceptual representation when reading L2 without necessarily going through the lexical pathway.

Noteworthily, with respect to intermediately proficient bilinguals, the RHM posits that there still exists a direct link between L1 and L2 representations that act as a cross-language pathway (Kroll and Stewart, 1994), which we call the lexical pathway. However, to the best of our knowledge, there have been few empirical experiments on the cross-language activation pathway in Mandarin–English bilinguals with intermediate English proficiency. Indeed, intermediate proficiency serves as a transitional stage between low and high levels of proficiency, garnering attention in the process of L2 acquisition (Kroll and de Groot, 2005; Schwieter, 2019). In a Spanish–Catalan bilingual study, intermediate bilinguals showed form (orthographical similarity of word) interference effects of L1 while performing the translation recognition task (Guasch et al., 2008), indicating direct activation from L2 to L1 lexical representation via the lexical pathway within these two alphabetic languages. However, such direct activation was also observed in the other two bilingual groups with lower and higher L2 proficiency. This finding contrasts with previous studies involving Mandarin–English bilinguals (Guo and Peng, 2003; Mo et al., 2005). Therefore, the aim of the present study was to examine the lexical pathway of L1 activation during L2 visual-word recognition in intermediate proficient Mandarin–English bilinguals.

With respect to the experimental paradigm, we found that previous studies tend to present both L1 and L2 to bilinguals. For instance, the cross-language priming task by Guo and Peng (2003) included both Chinese characters and English words. The previous empirical studies established that language mode significantly affects cross-lingual activation (Wang et al., 2009; Canseco-Gonzalez et al., 2010; Antoniou et al., 2012; Khachatryan et al., 2016; Wu et al., 2020; Yang et al., 2021b). Specifically, in the monolingual mode, the language not involved is barely activated in the bilingual whereas in the bilingual mode, both languages are activated (Grosjean, 2013; Khachatryan et al., 2016; Jiao et al., 2020). Considering this, we wondered whether cross-language activation through the lexical pathway in previous studies was affected by the language mode in their experimental paradigm. Therefore, in the following study, we will conduct experiments in the monolingual mode to examine the lexical pathway of cross-language activation from L2 to L1 lexical representations.

### 1.1 The present study

This study focuses on examining the lexical pathway for L1 activation during L2 visual-word recognition in Mandarin–English bilinguals with intermediate proficiency. According to the RHM, when bilinguals activate their L2 while reading their L1, they may go through either the conceptual pathway or the lexical pathway. Thus, to explore the potential existence of a direct lexical pathway from L2 to L1 lexical representations in intermediate-level Mandarin–English bilinguals, we must ensure that any such activation precedes, and does not arise via, the conceptual pathway. To achieve this goal, we need to design a task that prevents bilinguals from directly activating conceptual representation before examining the activation status of their L1 lexical representation.

During visual-word recognition, semantics are derived through the processing of the orthographic information of the words. In addition, previous research found that semantic priming can be controlled and even inhibited (Valdes et al., 2005; Spruyt et al., 2009). Specifically, if the individuals’ task is not relevant to semantic priming, an inhibitory control mechanism can be used to suppress this semantic activation (Mari-Beffa et al., 2005). Given that the automatic semantic priming can be modulated by feature-specific attention allocation (Spruyt et al., 2009), in Experiment 1, we designed a new visual-word recognition task, which we called “the vowel letter search task,” to focus the individual subjects’ attention on lexical characteristics instead of on semantics during L2 reading. This task requires participants to judge whether only one word of a word pair (priming word and target word), which appears successively, contains two adjacent vowel letters (e.g., “school,” “airport,” and “convenience” each contains two adjacent vowel letters). The participants should answer YES only if *either* the priming word *or* the target word has two adjacent vowel letters; they should answer NO if neither word nor both words contain two adjacent vowel letters. In addition, these word pairs, which were composed of priming words and target words, were either semantically related or unrelated.

Taken together, vowel letter condition (adjacent and non-adjacent) and semantic relatedness (related and unrelated) constitute two factors in Experiment 1, where vowel letter condition served as an explicit factor and semantic relatedness acted as an implicit factor. The combination of the two factors results in a total of four conditions (2 × 2), as shown in Table 1, S + V+, S + V−, S − V+, and S − V− (S+: semantically related, S−: semantically unrelated; V+: only one of the two words has two adjacent vowel letters, V−: otherwise). We examined whether the conceptual representation was activated by observing the effect of semantic relatedness. If the semantic relatedness of word pairs triggers a significant difference, we speculate that the conceptual representation might have been activated. If not, we speculate that it might not have been activated.

**Table 1:** Experimental design and stimuli examples of Experiment 1.

	Vowel letters adjacent		Vowel letters non-adjacent	
	S + V+		S + V−	
Semantically related	Physics	Science	Lemon	Banana
	物理	科学	柠檬	香蕉
	**S−V+**		**S−V−**	
Semantically unrelated	Story	airport	Cucumber	Rocket
	故事	机场	黄瓜	火箭

In Experiment 2, to create a monolingual mode that prevents bilinguals from activating lexical information from both their languages in parallel (Grosjean, 1998), we adopted the implicit priming paradigm, which only presents L2 with three factors, semantic relatedness (related and unrelated), character repetition in Mandarin (repetition and no repetition), and vowel letter condition (adjacent and non-adjacent). Among them, the vowel letter condition served as an explicit factor, while semantic relatedness and character repetition acted as implicit factors. With respect to the vowel letter condition, the task was designed to draw the participants’ attention to lexical information. The combination of the three factors results in a total of eight conditions (2 × 2 × 2), as shown in Table 2, S + R + V+, S + R + V−, S + R − V+, S + R − V−, S − R + V+, S − R + V−, S – R − V+, and S – R − V− (R+: character repetition in Mandarin, R−: no-repetition in Mandarin). During the experiment, we would first observe whether this task could prevent bilinguals from directly activating the conceptual representation before activating the L1 lexical representation. Then, we determined whether cross-language activation occurs via the lexical pathway by observing the bilinguals’ perception of character repetition in Mandarin.

**Table 2:** Experimental design and stimuli examples of Experiment 2.

	Character repetition				Character non-repetition			
	Vowel letters adjacent		Non-adjacent		Vowel letters adjacent		Non-adjacent	
	S + R + V+		S + R + V−		S + R − V+		S + R − V−	
Semantically related	Sky	Heaven	Television	Film	Peach	Strawberry	Pen	Ink
	天空	天堂	电视	电影	桃子	草莓	钢笔	墨水
	**S − R + V+**		**S − R + V−**		**S – R − V+**		**S – R − V−**	
Semantically unrelated	Turkey	Train	Princess	Company	Breakfast	Victory	Passenger	Sentence
	火鸡	火车	剬主	剬司	早餐	胜利	乘客	句子

Given that several previous behavioral measurement-based studies found the existence of only the conceptual pathway (Zeelenberg and Pecher, 2003; Li et al., 2009), we determined that if cross-language activation via the lexical pathway exists, it might be more implicit than activation via the conceptual pathway. Therefore, in the current study, in addition to behavioral measurement, we adopted the ERP approach to measure the temporal domain (e.g., reaction times) of brain activities. Additionally, we used N170, P200, and N400 as our primary analysis indices. These components are associated with distinct stages of word processing, focusing on activating word orthography (lexical representation) and meaning (semantic representation).

The N170 component, observed around 170 ms after presenting a linguistic stimulus, represents the early stage in visual language processing. N170 is associated with the early perception and recognition of word characteristics. It signifies the initial visual analysis of linguistic elements. In the present study, if the factor of character repetition in Mandarin triggers a significant main effect on the N170 amplitude, we would predict that Chinese has been activated at this early stage.

The P200 is a positively oriented waveform peaking approximately at 200 ms following the onset of external stimuli. This component typically manifests across the centro-frontal and parieto-occipital regions of the scalp. Previous studies showed that P200 was larger (more positive) for semantically related pairs relative to semantically unrelated pairs (Coulson et al., 2005; Hill et al., 2005; Landi and Perfetti, 2007; Wang et al., 2021). Thus, if the semantically related word pairs elicit a more positive P200 than the semantically unrelated word pairs in the present study, we hypothesized that semantics are engaged at this early stage. What is more, the P200 has been linked to the processing of orthographic features during the initial stage of lexical processing in Chinese (Lee et al., 2007; Kong et al., 2010). According to this, if the factor of character repetition in Mandarin triggers a significant main effect on the P200 amplitude, we would predict that Chinese has been activated at this stage.

N400 is a negative deflection peaking around 400 ms post-stimulus onset (Kutas and Hillyard, 1980; Yang et al., 2021a). This component was not only considered as an indicator for semantic priming, but we also observed it to be sensitive to repetition (Delogu et al., 2019; Tiedt et al., 2020; Pan et al., 2021; Yang et al., 2021b). As for semantic condition, individuals initiated a decreased N400 amplitude in response to semantic priming (Elston-Guttler et al., 2005; Kerkhofs et al., 2006). For instance, semantically unrelated word pairs (e.g., apple-desk) can trigger a more negative N400 amplitude than semantically related word pairs (e.g., wife–husband). With respect to repetition, if the objective (e.g., the first character in the word) is repeatedly shown, it will trigger a decreased N400 amplitude (Shimokochi, 1992; Yang et al., 2021b). To illustrate, the alliterative word pair 大人-大象 can trigger a decreased N400 amplitude than the pair 松鼠-桌子. In the current study, if the semantically unrelated word pairs elicit a more negative N400 than the semantically related word pairs, our hypothesis suggests the engagement of semantics at this early stage. Additionally, if the factor of character repetition in Mandarin triggers a significant main effect on the N400 amplitude, we would predict that Chinese has been activated at this stage.

### 1.2 Hypothesis

Overall, the design of the two experiments in the current study was guided by the following hypotheses:

*Hypothesis 1*: In Experiment 1, we hypothesized that the vowel letter search task presented in L2 prevents direct activation of a conceptual representation in the early stage.

During visual-word recognition, semantics are derived from the processing of the orthographic information of words. In addition, in a bilingual mental lexicon, the connection between the L2 lexical representation and the conceptual representation is weaker than that between the L1 lexical representation and the conceptual representation (Kroll and Stewart, 1994; Li et al., 2009). Given that semantic priming can be inhibited when an individual’s attention is directed to a low level of analysis like letters or sounds (Mari-Beffa et al., 2005; Valdes et al., 2005), we predicted that our designed vowel letter search task could achieve this goal of preventing semantic access in the early stage. Regarding the statistical results, if semantic relatedness did not show any significant difference, we speculate that the conceptual representation might not have been activated.

*Hypothesis 2*: In Experiment 2, we hypothesized that there exists a lexical pathway of cross-language activation from L2 lexical representation to L1 lexical representation in intermediate proficient bilinguals.

If the factor of character repetition in Mandarin produces significantly different results without semantic priming, it suggests that intermediate proficient bilinguals can activate their L1 through the lexical pathway, similar to observations in bilinguals with low proficiency (Guo and Peng, 2003). If the factor of character repetition in Mandarin does not show any significant difference, the lexical pathway of cross-language activation is non-existent in intermediate proficient bilinguals.

## 2 Experiment 1

### 2.1 Participants

A total of 24 Mandarin–English bilinguals (mean age = 23.17 years, range = 19–28 years old, seven males) comprised our effective participant pool for statistical analysis. They were right-handed with normal or corrected-to-normal vision. All the participants were born in mainland China and had no immigration experience. They all learned English in a classroom setting between the ages of 6 and 12 years. In their daily lives, Mandarin Chinese is used around 87.58% (SD = 7.45%) of the time, while English is used around 11.92% (SD = 7.32%) of the time. They reported their English proficiency level as intermediate. In the present study, participants’ language proficiency was assessed using the Oxford Placement Test (OPT) and a self-rating questionnaire. The OPT provided an objective measure of English (L2) proficiency (Jiao et al., 2020; Jiang et al., 2022), with higher scores indicating greater proficiency. The mean score of OPT of all participants was 34.21 (SD = 3.89). Each participant’s English proficiency was measured at an intermediate level (scores between 24 and 40 were classified as intermediate level). With respect to the subjective indicator of language proficiency, we asked participants to complete a self-reported questionnaire. They rated their proficiency in listening, speaking, reading, and writing for both their L1 and L2 on a 7-point scale (1 = not proficient, 7 = very proficient). On average, participants rated their L1 proficiency as follows: listening: 6.42 (SD = 0.83); speaking: 6.13 (SD = 0.74); reading: 6.46 (SD = 0.59); writing: 6.13 (SD = 0.74). For their L2, average ratings were as follows: listening: 4.46 (SD = 1.28); speaking: 4.25 (SD = 1.19); reading: 5.42 (SD = 1.06); writing: 4.92 (SD = 0.88). The results of paired-sample *t-*tests comparing the two languages were all significant [listening, *t* (23) = 9.21, *p* < 0.001; speaking, *t* (23) = 7.96, *p* < 0.001; reading, *t* (23) = 5.943, *p* < 0.001; writing, *t* (23) = 6.058, *p* < 0.001]. The results indicated that the participants in the experiment were Chinese-dominant bilinguals. All of them provided written informed consent and declared that they had no neurological or psychological impairments. After finishing the experiment, these participants received an agreed-upon amount of cash compensation for their participation.

### 2.2 Materials and design

An example of the stimuli in Experiment 1 we used is shown in Table 1. Each condition contains 40 English word pairs. To evaluate the semantic relevance of these word pairs, we used a five-point Likert scale with a separately recruited group of 15 bilingual participants. An ANOVA with semantic relatedness (related and unrelated) and vowel letter condition (adjacent and non-adjacent) as two factors were conducted for the semantic relevance of these word pairs. The result showed a significant main effect of relatedness [*F* (1, 156) = 663.941, *p* < 0.001, *η*^2^_p_ = 0.810], with the semantic relevance of the S+ word pairs significantly higher than the S− word pairs. No other main effect or interaction was significant (*p*s > 0.6). The word frequency of these words was obtained by using the new and improved word frequency database for British English (van Heuven et al., 2014). An ANOVA with semantic relatedness (related and unrelated), vowel letter condition (adjacent and non-adjacent), and order (priming word and target word) as three factors was conducted for the word frequency. No main effect or interaction was significant (*p*s > 0.4). In addition, the concreteness of the word pairs was also evaluated with a five-point Likert scale by a separately recruited group of 15 bilingual participants. An ANOVA with semantic relatedness in Chinese (related and unrelated), vowel letter condition (adjacent and non-adjacent), and order (priming word and target word) as three factors was also conducted for word concreteness. No main effect or interaction was significant (*p*s > 0.2). The word length (the number of letters) of these words was obtained from Microsoft Office Excel. An ANOVA was conducted with three factors: semantic relatedness in Chinese (related and unrelated), vowel letter condition (adjacent and non-adjacent), and order (priming word and target word) to analyze word length. No main effect or interaction was significant (*p*s > 0.4). During the experiments, the 80 word pairs were repeated twice, forming a total of 160 trials to ensure a sufficient number of measurements. These stimuli were presented pseudo-randomly.

### 2.3 Procedure

During Experiment 1, the participants were seated comfortably in an armchair at a distance of around 80 cm from the computer screen. All stimuli were displayed in the Times New Roman typeface with a size of 34, presented with E-Prime (2.0, Psychology Software Tools). The participants were instructed to judge rapidly whether only one word of a word pair (priming word and target word) contained two adjacent vowel letters and to indicate their answer by pressing a joystick button. The trial process was as follows: the fixation appeared in the center of the screen for 200 ms for attention; then, the priming word appeared in the center of the screen for 500 ms; after that, a blank replaced the screen of the priming word for a random period of time (500, 600, or 700 ms); finally, the target word appeared on the screen and did not disappear until the participants responded; after that, a blank screen appeared again for a random period of time (500, 600, or 700 ms). The trial process was in line with previously published studies (Thierry and Wu, 2007; Yang et al., 2021b).

### 2.4 Electroencephalogram recording and preprocessing

In Experiment 1, electroencephalography (EEG) data were recorded at a sampling rate of 500 Hz with a BrainAmp DC amplifier (Brain Products GmbH) and Recorder software (1.20, Brain Products GmbH) using a 62 Ag/AgCl electrodes elastic cap with an international 10/10 system (Easycap; Brain Products GmbH, Gilching, Germany), with the reference electrode placed at FCz and the ground electrode placed at AFz. Vertical and horizontal electrooculograms were also recorded for the elimination of ocular artifacts. Electrode impedance was maintained under 10 kΩ.

All EEG data were preprocessed using Brain Vision Analyzer software (Brain Products, Munich, Germany). This preprocessing contained re-referencing, EOG correction, filtering, segmentation, baseline correction, artifact rejection, and averaging. The EEG data were first re-referenced to the mathematically linked mastoids and then to the average of all EEG channels after the preprocessing. Then, ocular artifacts were corrected with the independent-component-analysis-based procedure embedded in the Brain Vision Analyzer software (Brain Products, Munich, Germany). Next, the EEG data were bandpass filtered offline from 0.1 to 35 Hz. The EEG from 200 ms before the onset of the target word to 800 ms after the onset was segmented into epochs (200 ms pre-target baseline).

The three components—N170, P200, and N400—along with their respective time windows, were determined by evaluating the mean global field power across the scalp. This measurement amalgamates contributions from all electrodes into a singular vector norm (Picton et al., 2000; Zhang et al., 2019). The time windows for N170, P200, and N400 were set at 150–200 ms, 150–300 ms, and 300–500 ms, respectively. Based on visual inspection of grand-averaged scalp topographies and existing literature on these components (Kong et al., 2010; Stuellein et al., 2016), the N170 component was identified at the electrodes Pz, P1, P2, POz, PO3, PO4, Oz, O1, and O2. P200 was observed at electrodes AF3, AF4, Fz, F1, F2, FCz, FC1, FC2, and Cz. N400 was detected at electrodes FCz, FC1, FC2, Cz, C3, C4, CPz, CP1, and CP2, aligning with previous relevant studies (Thierry and Wu, 2007; Martin et al., 2009). For statistical analysis, these components were investigated using the averaged ERP recorded at their corresponding electrodes.

### 2.5 Mixed-effects model analysis

Mixed-effects models were fitted in the R package lme4 (Bates et al., 2015). RTs and ERP data were submitted to a linear mixed-effects model. RTs were log-transformed to correct for skewed distribution.

Semantic relatedness was included in the models as the fixed factor and was sum coded (related = −0.5, unrelated = 0.5). For RTs, we started with a full model including the maximal random effects structure (Barr et al., 2013), i.e., random intercepts for participants and items and the random slope for semantic relatedness. For ERP analyses, we started with a full model, including random intercepts for participants and channels and the random slope for semantic relatedness. If the models failed to converge, we used a backward-stepping procedure until the model could be fitted. We conducted model comparisons to determine the best-fitting models. Specifically, the fitted models were compared to the random-intercepts-only models. The random-intercepts-only models were preferred if likelihood-ratio tests did not show a significant effect favoring the models with larger random effects structures; otherwise, the models with larger random effects structures were preferred. Additionally, we decided whether to include the random slope effects according to the Akaike Information Criterion (AIC) model comparisons of the models with and without these random slope effects. The model with the smallest AIC value (small indicates a better fit) was selected as the final model. The same selection procedures of the best-fitting model were applied to the subsequent analyses. For tests of fixed effects, the *p*-values were estimated with the package LmerTest (Kuznetsova et al., 2017).

### 2.6 Results

#### 2.6.1 Behavioral data

Both the ACC and the RT of Experiment 1 are shown in Table 2. The RTs’ analysis did not reveal a significant main effect of semantic relatedness (*E* = −0.007, *t* = −0.179, *p* = 0.858).

**Table 3:** Experimental design and stimuli examples of Experiment 2.

	Character repetition				Character non-repetition			
	Vowel letters adjacent		Non-adjacent		Vowel letters adjacent		Non-adjacent	
	S + R + V+		S + R + V−		S + R − V+		S + R − V−	
Semantically related	Sky	Heaven	Television	Film	Peach	Strawberry	Pen	Ink
	天空	天堂	电视	电影	桃子	草莓	钢笔	墨水
		**S − R + V+**		**S − R + V−**		**S – R − V+**		**S – R − V−**	
Semantically unrelated	Turkey	Train	Princess	Company	Breakfast	Victory	Passenger	Sentence
	火鸡	火车	剬主	剬司	早餐	胜利	乘客	句子

#### 2.6.2 Electrophysiological data

**Figure 1:**
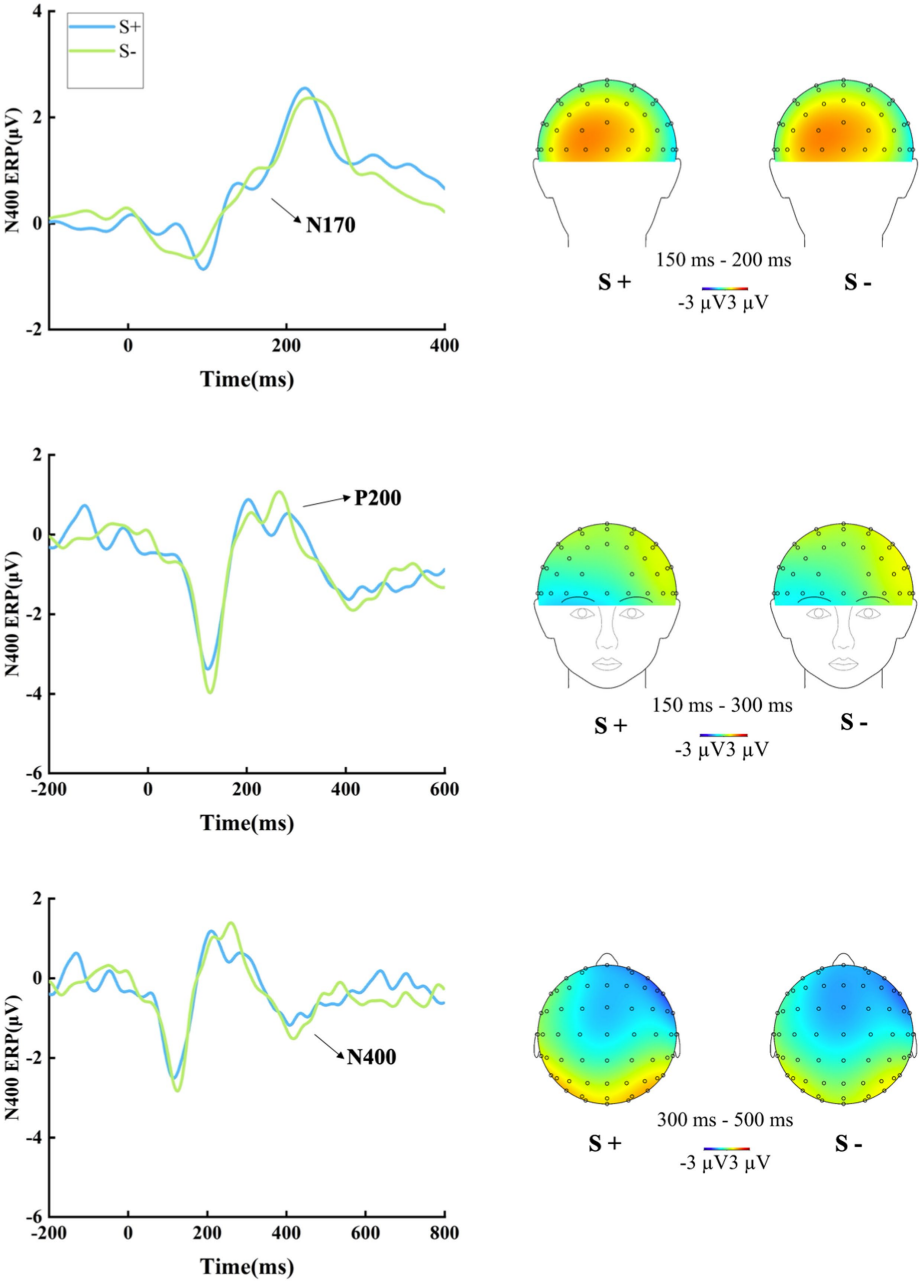
The grand-averaged ERP waves of the N170, P200, and N400 amplitudes, along with their topographic maps in Experiment 1. N170: The mixed-effects model on the N170 amplitudes did not reveal a significant main effect of semantic relatedness (*E* = 0.026, *t* = 0.103, *p* = 0.919). P200: The model on the P200 amplitudes did not show a significant main effect of semantic relatedness (*E* = 0.057, *t* = 0.403, *p* = 0.691). N400: The model on the N400 amplitudes yielded a non-significant main effect of semantic relatedness (*E* = −0.22, *t* = −0.725, *p* = 0.476).

The grand-averaged ERP waves of the N170, P200, and N400 amplitudes, along with their topographic maps in Experiment 1, are displayed in Figure 1.

In summary, neither the behavioral data nor the ERP results provided any evidence of semantic priming while participants performed our designed vowel letter search task for semantically related and unrelated word pairs. These Experiment 1 results align with Hypothesis 1, suggesting that our designed task, which encourages individuals to focus on the lexical characteristics of words, can effectively prevent semantic priming in the observed processing stage. Furthermore, these findings support the notion that semantic priming can be inhibited when individuals’ attention is directed to characteristics other than semantics (Mari-Beffa et al., 2005; Valdes et al., 2005; Spruyt et al., 2009).

## 3 Experiment 2

### 3.1 Participants

A total of 27 Mandarin–English bilinguals (mean age = 23.44 years, range = 19–28 years old, 12 males) comprised our effective participant pool for statistical analysis. They were right-handed with normal or corrected-to-normal vision. All the participants were born in mainland China and had no immigration experience. They had all learned English in a classroom setting between the ages of 4 to 10 years. In their daily lives, they use Mandarin Chinese around 88.11% (SD = 16.15%) of the time, while English is used around 12.42% (SD = 17.34%) of the time. They reported their English proficiency level as intermediate. In the present study, participants’ language proficiency was assessed using the OPT and a self-rating questionnaire. The OPT provided an objective measure of English (L2) proficiency (Jiao et al., 2020; Jiang et al., 2022), with higher scores indicating greater proficiency. The mean score of OPT of all participants was 34.59 (SD = 3.51). Each participant’s English proficiency was measured at an intermediate level. With respect to the subjective indicator of language proficiency, we asked participants to complete a self-reported questionnaire. They rated their proficiency in listening, speaking, reading, and writing for both their L1 and L2 on a 7-point scale (1 = not proficient, 7 = very proficient). On average, participants rated their L1 proficiency as follows: listening: 6.41 (SD = 0.83); speaking: 6.15 (SD = 0.85); reading: 6.59 (SD = 0.56); writing: 5.93 (SD = 0.81). For their L2, average ratings were as follows: listening: 4.48 (SD = 1.17); speaking: 3.96 (SD = 1); reading: 5.3 (SD = 1.21); writing: 4.48 (SD = 0.88). The results of paired-sample *t-*tests comparing the two languages were all significant [listening, *t* (26) = 8.083, *p* < 0.001; speaking, *t* (26) = 10.223, *p* < 0.001; reading, *t* (26) = 5.754, *p* < 0.001; writing, *t* (26) = 6.001, *p* < 0.001]. The results indicated that the participants in the experiment were Chinese-dominant bilinguals. All of them provided written informed consent and declared that they had no neurological or psychological impairments. After finishing the experiment, these participants received an agreed-upon amount of cash compensation for their participation.

### 3.2 Materials and design

An example of the stimuli under eight conditions in Experiment 2 is shown in Table 3. Each condition contains 15 English word pairs. As the vowel letter condition was only designed to draw the participants’ attention to lexical information, we did not consider this factor in our statistical analysis. The lexical characteristics of the stimuli in terms of semantic relatedness, word frequency, word concreteness, and translation consistency were controlled in a manner consistent with previous studies (Thierry and Wu, 2007; Khachatryan et al., 2016; Yang et al., 2021b).

To evaluate the semantic relevance of these word pairs, we used a five-point Likert scale with a separately recruited group of 15 bilingual participants. An ANOVA with semantic relatedness (related and unrelated) and character repetition in Mandarin (repetition and non-repetition) as two factors was conducted for the semantic relevance of these word pairs. The result showed a significant main effect of relatedness [*F* (1, 116) = 4124.094, *p* < 0.001, *η*^2^_p_ = 0.973], with the semantic relevance of the S+ word pairs significantly higher than the S− word pairs. In addition, there was also an interaction between semantic relatedness and character repetition in Mandarin [*F* (1, 116) = 5.448, *p* = 0.021, *η*^2^_p_ = 0.045]. The *t*-tests showed that the semantic relevance of S + R+ word pairs was significantly higher than the S − R+ word pairs [*t* (29) = 31.148, *p* < 0.001], the semantic relevance of S + R+ word pairs was significantly higher than the S − R− word pairs [*t* (29) = 38.3, *p* < 0.001], the semantic relevance of S + R− word pairs was significantly higher than the S − R+ word pairs [*t* (29) = 62.058, *p* < 0.001], the semantic relevance of S + R− word pairs was significantly higher than the S − R− word pairs [*t* (29) = 100.116, *p* < 0.001], the semantic relevance of S − R+ word pairs was significantly higher than the S − R− word pairs [*t* (29) = 2.414, *p* = 0.022], and the semantic relevance of S + R+ word pairs was similar to the S + R− word pairs [*t* (29) = −1.282, *p* = 0.210]. No other main effect or interaction was significant (*p*s > 0.9).

The word frequency of these words was obtained by using the new and improved word frequency database for British English (van Heuven et al., 2014). An ANOVA with semantic relatedness (related and unrelated), character repetition in Mandarin (repetition and non-repetition), and order (priming word and target word) as three factors was conducted for the word frequency. No main effect or interaction was significant (*p*s > 0.4). In addition, the concreteness of the word pairs was also evaluated with a five-point Likert scale by a separately recruited group of 15 bilingual participants. An ANOVA with semantic relatedness in Chinese (related and unrelated), character repetition in Mandarin (repetition and non-repetition), and order (priming word and target word) as three factors was also conducted for word concreteness. No main effect or interaction was significant (*p*s > 0.9). The word length of these words was obtained from Microsoft Office Excel. An ANOVA with semantic relatedness in Chinese (related and unrelated), character repetition in Mandarin (repetition and non-repetition), and order (priming word and target word) as three factors was also conducted for word length. No main effect or interaction was significant (*p*s > 0.6).

To ensure a level of consistency between the English words and their Chinese translations, another separately recruited group of 15 bilingual participants was required to determine whether the first Chinese translation that popped into their minds while they were reading an English word was the same as the Chinese translation presupposed in this study. Accordingly, each English word was assigned a score on the translation consistency represented by the proportion of people who would first think of the presupposed Chinese translation after viewing the English word. An ANOVA with semantic relatedness (related and unrelated) and character repetition in Mandarin (repetition and non-repetition) as two factors was conducted for the translation consistency score. No main effect or interaction was significant (*p*s > 0.1). During the experiments, the 120 word pairs were repeated twice, forming a total of 240 trials to ensure a sufficient number of measurements. These stimuli were presented pseudo-randomly.

### 3.3 Procedure

The procedure of Experiment 2 was the same as the procedure of Experiment 1.

### 3.4 EEG recording and preprocessing

The EEG recording and preprocessing of Experiment 2 was the same as the procedure of Experiment 1.

### 3.5 Mixed-effects model analysis

Mixed-effects models were fitted in the R package lme4 (Bates et al., 2015). RTs and ERP data were submitted to a linear mixed-effects model. RTs were log-transformed to correct the distribution skewness.

Semantic relatedness, Chinese character repetition, and their interaction were included in the models as fixed factors. Semantic relatedness and character repetition in Mandarin were sum coded (related = −0.5, unrelated = 0.5; unrepeated = −0.5, repeated = 0.5). For RTs and accuracy analyses, we started with a full model including random intercepts for participants and items and random slopes for semantic relatedness, Chinese character repetition, and their interaction. For ERP analyses, we started with a full model including random intercepts for participants and channels and random slopes for semantic relatedness, Chinese character repetition, and their interaction. For tests of fixed effects, the *p*-values were estimated with the package LmerTest (Kuznetsova et al., 2017). For statistically significant interactions, follow-up pairwise comparisons were made using the package emmeans (Lenth et al., 2022), with *p*-values corrected for multiple comparisons using Bonferroni. Cohen’s *d* effect sizes were calculated using the package EMAtools (Kleiman, 2021) for each fixed effect and the package psych (Revelle, 2022) for each pairwise comparison.

### 3.6 Results

#### 3.6.1 Behavioral data

Both the ACC and the RT of Experiment 2 are shown in Table 4. The RT analysis did not reveal a significant main effect of semantic relatedness (*E* = −0.041, *t* = −0.649, *p* = 0.518), character repetition in Mandarin (*E* = −0.022, *t* = −0.490, *p* = 0.625), or their interaction (*E* = −0.072, *t* = −0.806, *p* = 0.422).

**Table 4:** Statistics of ACCs and mean RT for participants in Experiment 2 in four factors.

		S + R+	S + R−	S − R+	S − R−
Experiment 2	ACC (%)	89.07	94.57	95.98	92.82
	RT (ms)	1425.324	1452.16	1338.598	1355.015

#### 3.6.2 Electrophysiological data

**Figure 2:**
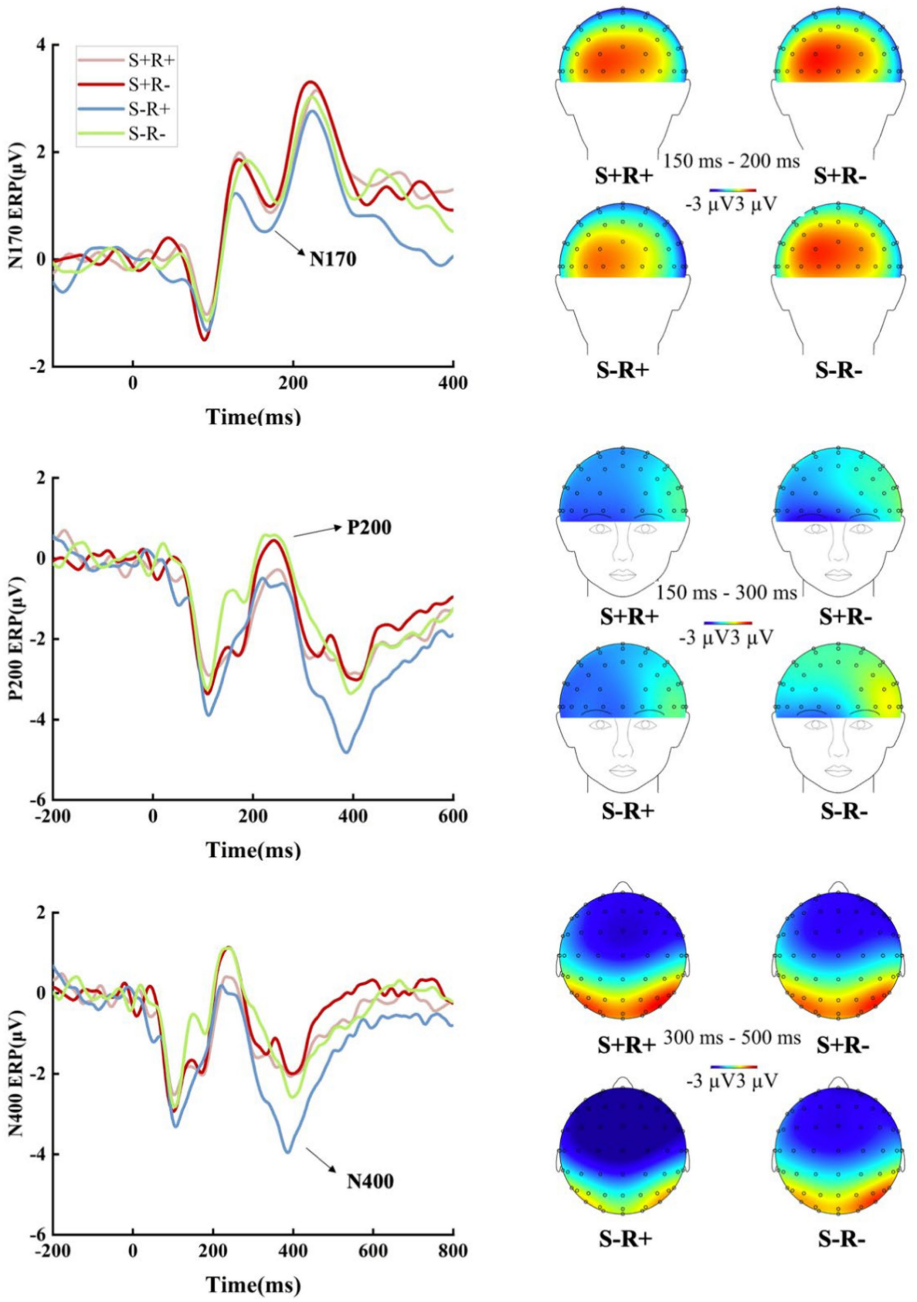
The grand-averaged ERP waves of the N170, P200, and N400 amplitudes, along with their topographic maps in Experiment 2. N170: The omnibus mixed-effects model on the N170 amplitudes did not reveal a significant main effect of semantic relatedness (*E* = −0.041, *t* = −0.649, *p* = 0.518), character repetition in Mandarin (*E* = −0.022, *t* = −0.490, *p* = 0.625), or their interaction (*E* = −0.072, *t* = −0.806, *p* = 0.422). P200: The model on the P200 amplitudes revealed a significant main effect of character repetition in Mandarin (*E* = −0.735, *t* = −3.375, *p* = 0.002), showing increased positivity in the unrepetitive condition compared to the repetitive condition. However, neither semantic relatedness (*E* = 0.345, *t* = 1.054, *p* = 0.301) nor the interaction between semantic relatedness and character repetition in Mandarin (*E* = −0.527, *t* = −0.956, *p* = 0.348) reached significance. N400: The model on the N400 amplitudes revealed a significant main effect of semantic relatedness (*E* = −0.764, *t* = −2.157, *p* = 0.040), showing increased negativity in the unrelated condition compared to the related condition. Critically, it also demonstrated a significant main effect of character repetition in Mandarin (*E* = −0.768, *t* = −2.071, *p* = 0.048), with increased negativity in the condition where characters were repeated compared to the non-repetitive condition. However, the interaction between semantic relatedness and character repetition in Mandarin failed to reach significance (*E* = −0.887, *t* = −1.192, *p* = 0.244).

The grand-averaged ERP waves of the N170, P200, and N400 amplitudes, along with their topographic maps in Experiment 2, are displayed in Figure 2.

To summarize, significant main effects of character repetition in Mandarin were observed within the P200 and N400 time windows, indicating the activation of bilinguals’ L1 lexical representation during these intervals. However, a significant main effect of semantic relatedness was only observed in the N400 time window. The absence of a main effect of semantic relatedness in the P200 time window suggests that bilinguals might activate their L1 lexical representation directly before engaging in conceptual representation.

## 4 Discussion

In the context of bilingual lexical activations between different languages, we still wondered, however, whether there might exist a direct activation pathway from L2 lexical representation to L1 lexical representation in intermediate proficient Mandarin–English bilinguals during visual-word recognition. Thus, our main purpose for conducting the current study was to explore this issue and, hopefully, use the results to make critical insights into the elaborate structure of bilingual mental lexicons. Specifically, in Experiment 1, we designed a new vowel letter search task on English word pairs. This task requires participants to judge whether only one word of a word pair contains two adjacent vowel letters. As a result, there was no evidence of semantic priming in either the behavioral data or the ERP results, including N170, P200, and N400, during task performance.

Then, in Experiment 2, we recruited Mandarin–English bilinguals to complete our vowel letter search task on English word pairs with occasional first character repetition between the Chinese counterparts of a word pair. Consistent with the findings of Experiment 1, we did not find any evidence supporting semantic priming in either the behavioral results or the N170 and P200 results. Notably, with respect to the factor of character repetition in Mandarin, we observed a significant main effect within both the P200 and N400 time windows, indicating the activation of bilinguals’ L1 lexical representation during these intervals. However, we failed to find a significant main effect of semantic relatedness in the P200 time window and only observed significance in the N400 time window. This indicates that conceptual representation was not activated in the early stage of L2 word processing. Taken together, these results suggest that bilinguals might directly activate their L1 lexical representation through the lexical pathway before engaging in conceptual representation.

The results of the present study supported our Hypothesis 1 that the vowel letter search task presented in L2 could prevent bilinguals from directly activating a conceptual representation in the early stage of word processing. In Experiment 1, although bilingual participants are presented with semantic-related word pairs, their behavioral and ERP results failed to show any significant effect of semantic priming. Similarly, in Experiment 2, the behavioral data and ERP results, specifically N170 and P200 components reflecting early word processing in bilingual participants, did not show any significant effects of semantic priming. Based on this, we inferred that conceptual representation might not be activated during the initial 300 ms processing stage after the onset of words in visual-word recognition. This finding is also consistent with previous viewpoint (Mari-Beffa et al., 2005; Valdes et al., 2005; Spruyt et al., 2009), suggesting that semantic priming can be controlled and even inhibited in the early stage of word processing.

The results of the present study also supported our Hypothesis 2 that there exists a lexical pathway of cross-language activation from L2 to L1 lexical representation for visual-word recognition in intermediate proficient bilinguals. In Experiment 2, although the behavioral data did not show any significant effect of character repetition in Mandarin, the P200 amplitude was significantly modulated by this factor without engagement of conceptual representation. Based on this, we inferred that intermediate proficient bilinguals could activate their L1 through the lexical pathway, which aligns with what has been observed in intermediate proficiency Spanish–Catalan bilinguals (Guasch et al., 2008).

Given the distinct significance of character repetition in Mandarin observed in both the behavioral results and the ERP results, we predict that the direct activation from L2 to L1 lexical representation via the lexical pathway in intermediate proficient bilinguals is implicit. Our results differ from those of Guo and Peng (2003) in Mandarin–English bilinguals with low proficiency. In their study, direct activation from L2 to L1 lexical representation was explicit rather than implicit, as evidenced by the significant behavioral result indicating a strong connection between L2 lexical representation and L1 lexical representation. Our results also contrast with those of Mo et al. (2005) in highly proficient Mandarin–English bilinguals. In their study, the result of such activation was insignificant, suggesting that direct activation from L2 to L1 lexical representation did not exist. Taken together, we infer that the level of proficiency in L2 modulates the strength of the lexical pathway of activation between L2 lexical representation and L1 lexical representation. That is, the more proficient the bilinguals’ L2 is, the weaker the strength of the lexical pathway becomes.

Our findings, together with the studies by Guo and Peng (2003) and Mo et al. (2005), provide firm support for the RHM in the context of Mandarin–English bilinguals. The model posits that lexical representations from the two languages are connected to each other and also to the conceptual representation. In addition, the strength of these connections varies based on language direction and proficiency in L2. In particular, the model suggests that in the early stages of L2 learning, the connection between L2 lexical representation and conceptual representation is very weak whereas the connection between L1 lexical representation and conceptual representation is always strong when these bilinguals read their L2, they comprehend by resorting to their L1 lexical representation. Guo and Peng’s (2003) study reveals direct activation from L2 to L1 representation in low-proficiency bilinguals, supporting the model at the initial stage. As proficiency increases, the connections between L2 lexical representation and L1 lexical representation and the conceptual representation develop and strengthen, while the connection between L2 lexical representation and L1 lexical representation becomes weaker. In our present study, implicit activation in intermediate bilinguals supports this description at this stage. With very high proficiency, direct access to the conceptual representation could be achieved from L2 lexical representation, and the connection between L2 lexical representation and L1 lexical representation almost disappears. Mo et al.’s (2005) study supports this model at this stage by indicating the absence of direct activation between L1 and L2 representation.

Comparing the insignificant N400 effect of semantic relatedness in Experiment 1 and the significant N400 effect of semantic relatedness in Experiment 2, we predicted that the semantic priming in Experiment 2 might be triggered by character repetition in Mandarin. Although semantic priming appeared in Experiment 2, this did not necessarily mean that the L1 of the bilinguals was activated via the conceptual pathway during L2 visual-word recognition, as semantic priming occurred only with the engagement of character repetition in L1. In other words, semantic priming appeared after bilinguals perceived the character repetition in their L2. To explain why character repetition in L1 could prompt further semantic priming, we introduced the Spreading-Activation Theory of Semantic Processing (Collins and Elizabeth, 1975) for further analysis. This theory posits that the brain’s vocabulary memory consists of conceptual networks and lexical networks. When an individual perceives a word, its signal is activated along the network pathway with a decreasing gradient, resembling a signal from a source that gradually decays or even disappears as it spreads out. Additionally, if the external stimulus is a variable, and the sum of the intersections for the variable activated in the networks reaches a threshold, the brain may re-evaluate these signals in the network and continue to activate related information. Based on this theory, we speculate that bilingual participants in this study could activate conceptual representation while performing the vowel letter search task because the factor of character repetition in L1 might act as a variable for them and stimulate their brain reactivation.

As for the pathway to activate the conceptual representation, our result is different from the result by Martin et al. (2009). In the present study, the conceptual representation activation is triggered by the factor of Mandarin character repetition in L1 lexical representation. In Martin et al.’s (2009) study, however, the conceptual representation activation is triggered by their L2 representation directly. In Martin et al.’s (2009) study, the letter counting task was used as the main experimental task to redirect early bilinguals’ attention away from the semantic content of the stimuli. These early bilinguals had acquired their languages in parallel from birth. In a letter counting task, the individual participants were presented with words and required to make word length decisions on words [e.g., indicating whether alphabetic language words had (a) less than five letters or (b) five or more than five letters]. The organization of the word stream in pairs were either semantically related or unrelated. As a result, these semantically unrelated word pairs triggered a significant N400 amplitude compared to semantically related word pairs, implying that the conceptual representation was activated in this group of bilinguals.

The divergence might be attributed to two factors: different tasks and variations in L2 proficiency levels. Concerning the task, the letter counting task requires bilinguals to judge only one word at a time, while the vowel letter search task requires bilinguals to consider a word pair at a time. Thus, compared with the word length decision task, the vowel search task is more complex, requiring greater attentional resources for judgment, leaving little spare capacity for semantic processing. With respect to the variations in L2 proficiency levels, the early Welsh–English bilinguals in Martin et al.’s (2009) study were likely to be highly proficient, while the bilingual participants in the present study were not early bilinguals and were evaluated to have intermediate proficiency. According to the RHM, the link between the L2 lexical representation and the conceptual representation is weaker than that between the L1 lexical representation and the conceptual representation. Moreover, the more proficient one becomes in their L2, the stronger the link between the L2 lexical representation and the conceptual representation. Based on these, we speculated that the link between the L2 lexical representation and the conceptual representation for the early bilingual participants by Martin et al. (2009) was stronger than that in our present study—this is what led to the different results of conceptual representation activation during L2 reading.

The current study has three potential limitations that need to be addressed in future studies. First, since the current study aimed to explore the lexical pathway between the L2 lexical representation and the L1 lexical representation, we only required participants to perform our designed vowel letter search task, without considering the conceptual pathway. The latter was repeatedly examined by asking participants to perform semantic relatedness judgments (Thierry and Wu, 2004, 2007; Wu and Thierry, 2010). In our experiment, we only required participants to perform our designed vowel letter search task. Second, since the study by Thierry and Wu (2007) established that native Mandarin speakers with repeated characters for semantically related and unrelated word pairs revealed that the repeated characters triggered a decrease in N400 in both conditions, we did not repeat this experiment. We only focused our attention on the cross-language phenomenon for bilinguals by observing the implicit factor, the Chinese character repetition after translation. Finally, we only considered bilinguals whose L1 was logographic and L2 was alphabetic. For bilinguals whose L1 and L2 were alphabetic writing systems, what would be their results if they performed similar vowel letter search tasks at an intermediate L2 level? If we invited these bilinguals to conduct such a study, it might help to find out how our study differs from Martin’s.

## 5 Conclusion

In the present study, we utilized both behavioral measurement and ERP technology to demonstrate the existence of the lexical pathway for the automatic activation of L1 during L2 visual-word recognition in intermediate proficient bilinguals. Our findings, along with studies on Mandarin–English bilinguals with low and high proficiency, indicate that the level of proficiency in L2 modulates the strength of the activation pathway between L2 lexical representation and L1 lexical representation. Taken together, this evidence provides firm support for the RHM in the context of Mandarin–English bilinguals. Specifically, the more proficient the bilinguals are in L2, the weaker the strength of the activation pathway. How these activation pathways might function in different contexts and whether they vary for bilinguals with different language backgrounds are intriguing topics for future research.

## Data availability statement

The raw data supporting the conclusions of this article will be made available by the authors, without undue reservation.

## Ethics statement

The studies involving humans were approved by the Ethics Committee of the School of Psychology at Tsinghua University. The studies were conducted in accordance with the local legislation and institutional requirements. The participants provided their written informed consent to participate in this study.

## Author contributions

SY: Conceptualization, Data curation, Formal analysis, Investigation, Methodology, Writing – original draft, Writing – review & editing. SJ: Data curation, Formal analysis, Writing – review & editing. MJ: Data curation, Funding acquisition, Methodology, Project administration, Resources, Software, Supervision, Validation, Writing – review & editing. QG: Writing – review & editing.
